# Lichen-derived caperatic acid and physodic acid inhibit Wnt signaling in colorectal cancer cells

**DOI:** 10.1007/s11010-017-3178-7

**Published:** 2017-09-08

**Authors:** Jarosław Paluszczak, Robert Kleszcz, Elżbieta Studzińska-Sroka, Violetta Krajka-Kuźniak

**Affiliations:** 1 Department of Pharmaceutical Biochemistry, Poznan University of Medical Sciences, ul. Święcickiego 4, 60-781 Poznan, Poland; 2 Department of Pharmacognosy, Poznan University of Medical Sciences, ul. Święcickiego 4, 60-781 Poznan, Poland

**Keywords:** Depsides, Depsidones, Physodic acid, Caperatic acid, Wnt pathway, Colorectal cancer

## Abstract

Lichens are a source of secondary metabolites which possess important biological activities, including antioxidant, antibacterial, anti-inflammatory, and cytotoxic effects. The anticancer activity of lichens was shown in many types of tumors, including colorectal cancers (CRC). Several studies revealed that the application of lichen extracts diminished the proliferation of CRC cells and induced apoptosis. Colon carcinogenesis is associated with aberrations in Wnt signaling. Elevated transcriptional activity of β-catenin induces cell survival, proliferation, and migration. Thus, the inhibition of Wnt signaling is a promising therapeutic strategy in colorectal cancer. The aim of this study was the evaluation of the effects of lichen-derived depsides (atranorin, lecanoric acid, squamatic acid) and depsidones (physodic acid, salazinic acid) and a poly-carboxylic fatty acid—caperatic acid, on Wnt signaling in HCT116 and DLD-1 colorectal cancer cell lines. HCT116 cells were more sensitive to the modulatory effects of the compounds. PKF118-310, which was used as a reference β-catenin inhibitor, dose-dependently reduced the expression of the classical β-catenin target gene—*Axin2* in both cell lines. Lecanoric acid slightly reduced *Axin2* expression in HCT116 cells while caperatic acid tended to reduce *Axin2* expression in both cell lines. Physodic acid much more potently decreased *Axin2* expression in HCT116 cells than in DLD-1 cells. Physodic acid and caperatic acid also diminished the expression of *survivin* and *MMP7* in a cell line and time-dependent manner. None of the compounds affected the nuclear translocation of β-catenin. This is the first report showing the ability of caperatic acid and physodic acid to modulate β-catenin-dependent transcription.

## Introduction

Lichens, which are symbiotic organisms consisting of fungi and photosynthetic partners (algae and/or cyanobacteria), are a source of a broad group of unique secondary metabolites, many of which possess important biological activities [1, 2]. One of the best studied lichen-derived compounds is usnic acid, however, there is abundance of other compounds which have not been studied in greater detail so far. Investigations using lichen extracts prevail and data describing the action of single compounds are limited. More importantly, the molecular mechanisms of the observed action of both lichen extracts and single compounds remain largely unknown. Lichen-derived extracts and compounds have been shown to exert antioxidant, antibacterial, antiviral, antipyretic, anti-inflammatory, and cytotoxic activity [2]. Some lichen extracts show selective cytotoxicity towards cancer cells, leaving non-cancerous cells largely unaffected [3]. Lichen compounds may exert anticancer properties due to the ability to induce cell cycle arrest and apoptosis [4–6]. However, this frequently requires the application of high concentrations of the chemicals.

Depsides (e.g. atranorin) and depsidones (e.g. physodic acid) are synthesized by lichens in the acetyl-polymalonyl pathway and they also have been pointed as possessing a promising pharmacological profile. Most of the studies conducted so far indicate the antioxidant, antimicrobial, and cytotoxic effects of these compounds [7–11]. On the other hand, there is some evidence that these compounds may modulate cell function by affecting signaling pathways. In this regard, it has been recently shown that atranorin and lecanoric acid may inhibit AhR-XRE-dependent gene transcription [12].

The anticancer activity of lichens was shown in many types of tumors, including colorectal cancers (CRC). Colorectal cancer is a major health burden leading to high morbidity and mortality. Several studies revealed that the application of lichen extracts diminished the proliferation of CRC cells and induced apoptosis [8, 9, 13]. Moreover, the use of lichen extracts may decrease tumor burden in animal CRC models [14]. It is widely accepted that the initiation and progression of CRC is significantly associated with aberrations in Wnt signaling [15]. Around half of CRC cases show inactivating mutations of *APC* tumor suppressor, which is one of the most important negative regulators of the Wnt pathway. Activating mutations in *CTNNB1* gene, which encodes β-catenin, and also of other genes, may be another reason for the enhancement in Wnt signaling. The increased transcriptional activity of β-catenin induces cell survival, proliferation, and migration by stimulating the expression of such target genes as *CCND1*, *c*-*MYC*, *BIRC5* (*survivin*), *MMP*-*2*, *MMP*-*7*, *MMP*-*9* [16]. Thus, the inhibition of Wnt signaling is one of the important pharmacological targets in the treatment of colorectal tumors [17, 18]. Given the anticancer activity of lichen compounds in CRC, it is interesting whether these effects are mechanistically related to the modulation of canonical Wnt signaling, which is the most commonly upregulated pathway in CRC.

The aim of this study was the evaluation of the effects of depsides (atranorin, lecanoric acid, squamatic acid) and depsidones (physodic acid, salazinic acid) and a poly-carboxylic fatty acid—caperatic acid, which were derived from different lichen species, on the Wnt signaling in colorectal cancer cell lines. To the best of our knowledge, the biological activity of caperatic acid has not been studied so far. The results of the study indicate that physodic acid and caperatic acid have the ability to down-regulate the transcription of β-catenin-dependent genes.

## Materials and methods

### Preparation of lichen compounds

**Fig. 1:**
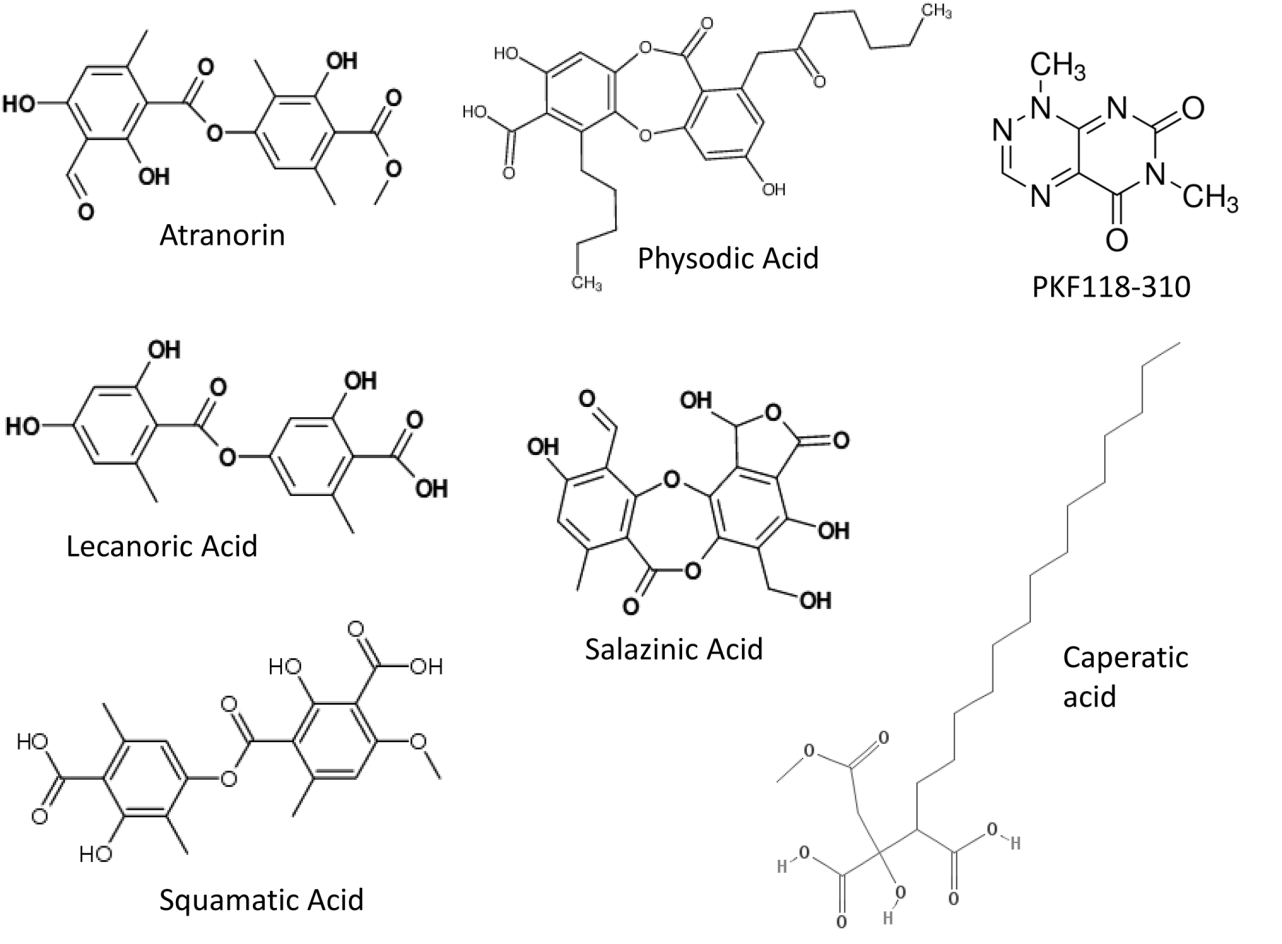
The chemical structure of the investigated lichen compounds and β-catenin inhibitor PKF118-310

The lichen specimens (*Platismatia glauca*, *Cladonia uncialis*, *Parmelia sulcata*, *Hypogymnia physodes,* and *Hypocenomyce scalaris*) were collected in September 2013 in the Notec Primeval Forest, Poland and authenticated by Dr. Daria Zarabska-Bożejewicz (The Institute for Agricultural and Forest Environment of the Polish Academy of Sciences in Poznan). Voucher specimens (ES 2013.01–ES 2013.05) have been deposited in the herbarium of the Department of Pharmacognosy of Poznan University of Medical Sciences. Air-dried lichen thallus was coarsely crushed (10 g for: *P. glauca*, *C. uncialis*, *P. sulcata*, *H. physode*s; 2 g for *H. scalaris*). Each sample was extracted by shaking at room temperature using solvents with increasing polarity. All the samples were extracted four times with 150 mL (600 mL total) of hexane (POCH, Poland) and then five times with 150 mL (750 mL total) of diethyl ether (POCH, Poland) (*P. glauca*) or of acetone (POCH, Poland) (the remaining samples), successively, each time for 1 h. The extracts were filtered using Whatman filter paper No. 1 and then the fractions obtained by one solvent were concentrated under the reduced pressure at 35 °C. Different methods of isolation were applied to provide the desired lichen substances. Atranorin (5 mg) (*P. glauca*) and squamatic acid (30 mg) (*C. uncialis*) were obtained by spontaneous crystallization during concentration of the hexane or acetone extract, respectively. Salazinic acid (15 mg) was isolated by recrystallization of the acetone extract of *P. sulcata* (30 mg) from the acetone:water (8:2) mixture. The isolation of physodic acid (6 mg) from the acetone extract of *H. physodes* (100 mg) and caperatic acid (35 mg) from diethyl ether extract of *P. glauca* (100 mg) were carried out applying silica column chromatography (diameter and length of filling—1.5 × 8 cm, silica gel 230–400 mesh, Sigma-Aldrich, USA) using the increasing gradient of mixtures of solvents (toluene-ethyl acetate 110:0 to 100:10 for physodic acid and hexane–ethyl acetate 100:0 to 60:40 for caperatic acid). Lecanoric acid (5 mg) was obtained from the acetone extract of *H. scalaris* (17 mg) using preparative thin layer chromatography (PLC 60 *F*_254_, 2 mm, Merck, Germany; solvent: diethyl ether-glacial acetic acid 100:1, ethyl acetate was utilized to wash the silica gel). The chemical structure of the compounds was identified by UV, ^1^H NMR, ^13^C NMR, and MS analysis and the obtained data were comparable to the published values [19]. The chemical structures of the studied compounds are presented in Fig. 1.

### Cell culture and viability assay

The HCT116 and DLD-1 colorectal carcinoma cell lines were purchased from ECACC and the HaCaT cell line of spontaneously immortalized keratinocytes was purchased from Cell Lines Service (Germany). The cells were grown in Dulbecco’s Modified Eagle’s Medium (DMEM, Biowest SAS, France) with the addition of 10% FBS (EURx, Poland) and 1% antibiotics solution (penicillin and streptomycin) (Sigma-Aldrich, USA) at 37°C in 95% humidified and 5% CO_2_ atmosphere.

The effect of the tested lichen compounds on the viability of HCT116, DLD-1, and HaCaT cells was assessed by the MTT assay according to a standard protocol. Cells (10^4^) were seeded in wells of a 96-well plate and after 24 h of pre-incubation in DMEM supplemented with 5% FBS and antibiotics, the tested compounds were added to the culture medium in various concentrations and the cells were further incubated for 48 h. Then, cells were washed with PBS buffer, and incubated for 4 h in the presence of fresh medium containing MTT salt (0.5 mg/ml). Afterwards, acidic isopropanol was added into wells in order to dissolve formazan crystals and absorbance was measured at 570 and 690 nm. All the experiments were repeated three times with at least four measurements per assay.

### Cell cycle analysis

The analysis of cell cycle was performed using Muse Cell Cycle Kit (Merck, Germany) according to the manufacturer’s protocol. Briefly, cells were grown for the indicated time in the presence of the tested compounds (PKF118-310, caperatic acid, physodic acid) or camptothecin (a positive control) and upon termination of incubation cells were fixed in 70% ethanol and stored at −20 °C for at least 24 h. Subsequently, cells were washed with PBS buffer and stained with propidium iodide in the presence of RNase A. Next, samples were analyzed by flow cytometry on Muse Cell Analyzer and data were analyzed using Muse 1.5 Analysis software. All the experiments were repeated twice.

### The analysis of apoptosis

The externalization of phosphatidylserine was used as a marker of apoptosis and was analyzed by Annexin V staining (Muse Annexin V & Dead Cell Kit; Merck, Germany) according to the manufacturer’s protocol. 7-AAD stain was also used to differentiate between early and late apoptotic cells. Briefly, cells were grown for the indicated time in the presence of the tested compounds (PKF118-310, caperatic acid, physodic acid) or camptothecin (a positive control) and upon termination of incubation cells were collected and immediately stained with Annexin V and 7-AAD. Next, samples were analyzed by flow cytometry on Muse Cell Analyzer and data were analyzed using Muse 1.5 Analysis software. All the experiments were repeated twice.

### Isolation of total RNA and cDNA synthesis

2 × 10^6^ cells were seeded in 100 mm culture dishes and after 24 h of pre-incubation in DMEM containing 5% FBS cells were treated with lichen compounds at the concentration of 50 μM with the exception of physodic acid which was used at the concentration of 25 μM. A small-molecule β-catenin inhibitor PKF118-310 was used as a positive control at two concentrations—0.2 and 0.3 μM. Control cells were treated with the vehicle only (DMSO). After the incubation (24 or 48 h) total RNA was isolated using Universal RNA Purification Kit (EURx, Poland) and subsequently subjected to reverse transcription using RevertAid First Strand cDNA Synthesis Kit (Thermo Fisher Scientific, USA), according to manufacturer’s protocol. All the experiments were repeated twice.

### Quantitative real-time PCR

Quantitative real-time PCR was performed using Hot FIREPol EvaGreen qPCR Mix Plus (Solis Biodyne, Estonia) and LightCycler 96 (Roche, Germany). Primer sequences are listed in Table 1. Primers were designed using Beacon Designer software and BLAST searched to minimize unspecific binding. Only primer pairs generating intron-spanning amplicons were selected. All the oligonucleotides were synthesized at the Institute of Biochemistry and Biophysics, Warsaw, Poland. All qPCR reactions were run in triplicate. The protocol started with 15 min enzyme activation at 95 °C, followed by 40 cycles of 95 °C for 15 s; 56°C for 20 °s; 72 °C for 40 s and the final elongation at 72 °C for 5 min which was followed by melting curve analysis in order to confirm the generation of a single amplification product. As previously described [20], the mean expression of the TATA-box binding protein (*TBP*) and porphobilinogen deaminase (*PBGD*) was used for data normalization and the ΔΔ*C*_t_ method was used for fold-change calculation.

**Table 1:** The sequence of starters used in qPCR reactions

Gene	Sequence 5′ → 3′	Amplicon size (bp)
*Axin2*	*forward* TAGGTTCTGGCTATGTCTTTGC *reverse* GCCTTCACACTGCGATGC	175
*BIRC5*	*forward* GGACCACCGCATCTCTAC *reverse* CCTTGAAGCAGAAGAAACAC	143
*CCND1*	*forward* CCCTCGGTGTCCTACTTC *reverse* TCCTCGCACTTCTGTTCC	107
*CTNNB1*	*forward* GGTGACAGGGAAGACATC *reverse* GACAAAGGGCAAGATTTCG	199
*c*-*Myc*	*forward* TTACAACACCCGAGCAAG *reverse* AATCCAGCGTCTAAGCAG	133
*MMP7*	*forward* GCAGTGATGTATCCAACCTATG *reverse* GCAACAATGATATACAATCCAATG	172

### Preparation of cytosolic and nuclear fractions

Subcellular extracts were prepared using the Nuclear/Cytosol Fractionation Kit (BioVision, USA) according to the manufacturer’s protocol. Protein concentration was assessed with the Lowry assay and then the samples were stored at −80 °C until further analysis.

### Western blot assay

The content of β-catenin, phospho-β-catenin (Thr41/Ser45), and Axin2 in cellular extracts was assessed using the Western blot technique. Cytosolic (β-catenin, phospho-β-catenin, Axin2) or nuclear (β-catenin) extracts were separated on 7.5% SDS-PAGE gels (Bio-Rad, USA) and transferred onto nitrocellulose membrane. After blocking with 10% skimmed milk, the membranes were incubated with primary rabbit polyclonal antibodies (Santa Cruz Biotechnology, USA) directed against β-catenin, phospho-β-catenin or Axin2. The analysis of β-actin or lamin A served as a loading control. After washing, the membranes were probed with alkaline phosphatase-labeled secondary antibodies (anti-rabbit IgG, Santa Cruz Biotechnology, USA) and stained using the BCIP/NBT AP Conjugate Substrate Kit (Bio-Rad, USA). The Quantity One software was used to determine the amount of the immunoreactive products and the values were calculated as relative absorbance units (RQ) per mg protein.

### Cell migration assay

Cells were seeded (5 × 10^5^/well) in a 24-well plate and grown overnight to confluence. A scratch was performed using a 10 μl tip and wells were washed with PBS buffer in order to get rid of detached cells. Fresh medium containing the indicated concentrations of the tested compounds was subsequently added to wells and photographs were immediately taken using JuLI FL microscope (NanoEntek, Korea). Cells were further grown for 24 h and wells were photographed again at the same areas. Area covered by cells (%) was assessed using JuLI FL software and the difference in the coverage of the growth area by cells between the two time points was calculated for each well. The experiment was repeated twice with three independent replicates per each assay. The relative effect of the tested compounds on cell migration was calculated by comparing the mean difference in cell coverage area between compound-treated cells and vehicle control (test [area_24_−area_0_]/control [area_24_−area_0_]).

### Invasion assay

Changes in the cell invasive potential were assessed using the fluorometric CytoSelect Cell Invasion Assay (Cell Biolabs, USA) according to the manufacturer’s protocol. Briefly, 1.5 × 10^5^ HCT116 cells were seeded in DMEM containing 1% antibiotics solution (lacking FBS) into the basement membrane inserts which were placed in wells filled with DMEM containing 10% FBS and 1% antibiotics solution. The cells were incubated in the presence of 0.3 µM PKF118-310, 50 µM caperatic acid, 25 µM physodic acid, or vehicle for 48 h. Then, invading cells were detached from the basement membrane, lysed and quantified using CyQuant GR fluorescent dye (480/520 nm). The experiments were repeated three times.

### Statistical analysis

The statistical significance between the experimental groups and their respective controls was assessed using Student’s *t* test with *p* ≤ 0.05 considered as significant.

## Results

### Cell viability analysis

**Fig. 2:**
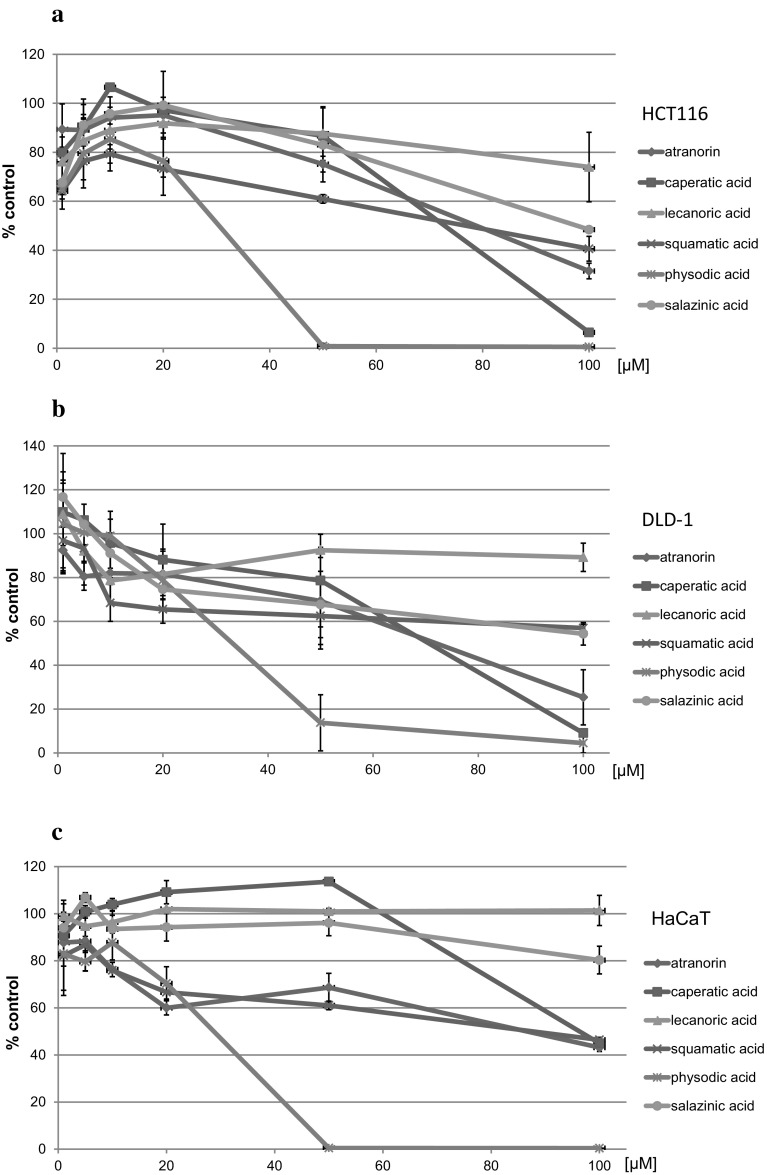
The effect of lichen compounds on the viability of HCT116 (**a**), DLD-1 (**b**), and HaCaT (**c**) cells. Mean values ± SEM from three independent experiments are shown

The effects of the tested lichen compounds on the viability of colorectal cancer cells were assessed using the MTT assay. The results are shown in Fig. 2. Physodic acid exerted the strongest cytotoxic activity in both HCT116 and DLD-1 cells leading to a very significant reduction in cell number at 50 μM concentration. Caperatic acid showed strong cytotoxicity at 100 μM, similar to atranorin. The other compounds exerted moderate cytotoxic effects at the concentration of 100 μM. Therefore, the modulatory activity of lichen compounds was further tested at the concentration of 50 μM while a lower concentration was used in the case of physodic acid (25 μM) because of its higher cytotoxicity.

In order to verify whether the cytotoxic effects of the studied compounds are specific towards cancer cells, we tested their influence on the HaCaT immortalized keratinocyte cell line (Fig. 2c). In general, HaCaT cells were less sensitive to the cytotoxic effects of the compounds with the exception of physodic acid and squamatic acid, which exerted a cytotoxic response similar as in colorectal cancer cell lines.

### The effect of lichen compounds on the expression of β-catenin target genes

**Fig. 3:**
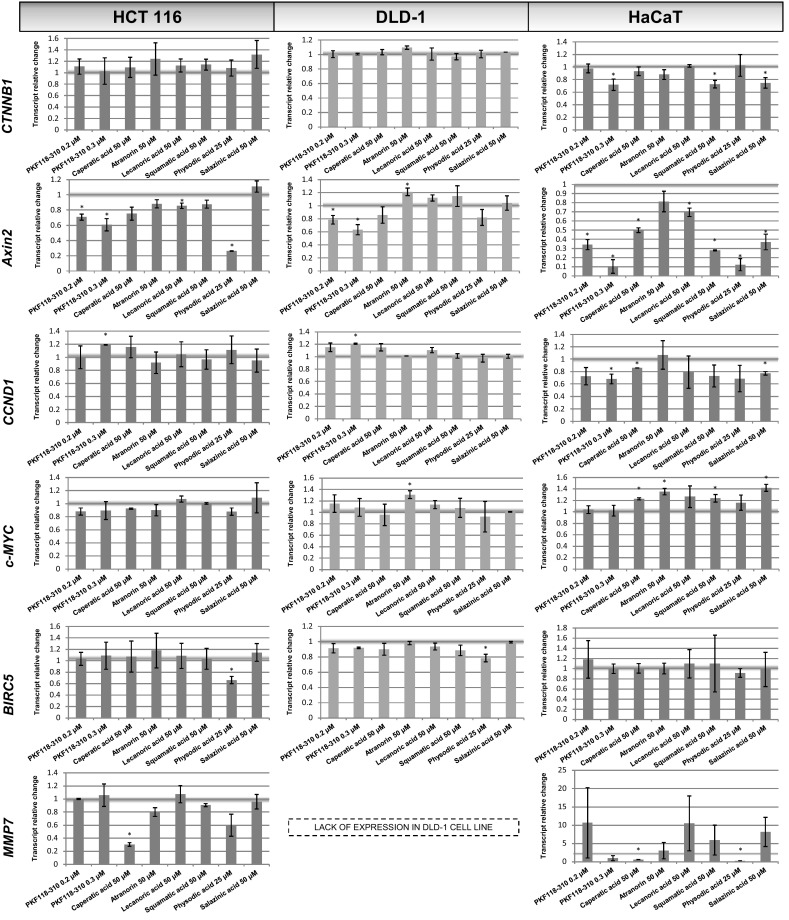
The effect of lichen-derived chemicals and PKF118-310 on the expression of *CTNNB1* and β-catenin target genes (*Axin2, CCND1, c*-*MYC, BIRC5, MMP7*) in HCT116, DLD-1, and HaCaT cell lines after 48 h incubation. The level of each transcript was calculated in relation to cells treated with the vehicle where expression was equal 1. Mean values ± SD from two independent experiments are shown. Asterisk above bars denotes statistically significant changes, *p* ≤ 0.05

Next, we investigated whether the chemicals are capable of modulating the expression of genes regulated transcriptionally by β-catenin (*Axin2*, *CCND1*, *c*-*MYC*, *MMP7*, *BIRC5*). The cells were grown in the presence of the compounds for 48 h before the isolation of RNA. The inhibitor of the interaction between β-catenin and TCF4 (PKF118-310) was used as a positive control. The results are shown in Fig. 3. In general, HaCaT cells showed most significant changes while HCT116 cells were slightly more sensitive to the modulatory effects of the compounds than DLD-1 cells. PKF118-310 dose-dependently reduced the expression of the classical β-catenin target gene—*Axin2* in all three cell lines. Physodic acid potently decreased *Axin2* expression in HCT116 cells, however, the effects in DLD-1 cells were much weaker and not statistically significant. Also, caperatic acid tended to reduce *Axin2* expression in both colorectal cancer cell lines, although the changes were not statistically significant. Lecanoric acid slightly reduced *Axin2* expression in HCT116 cells. On the other hand, all lichen compounds downregulated the expression of *Axin2* in HaCaT cells. Additionally, caperatic acid, and salazinic acid reduced the level of *CCND1* in HaCaT cell line. Moreover, physodic acid reduced the expression of *BIRC5* which encodes survivin in both colorectal cancer cell lines and also tended to decrease the level of *MMP7* transcript in HCT116 cells. On the other hand, caperatic acid led to an even stronger reduction in *MMP7* expression in HCT116 cells while in HaCaT cells both caperatic acid and physodic acid led to a strong reduction in the level of *MMP7*.

**Fig. 4:**
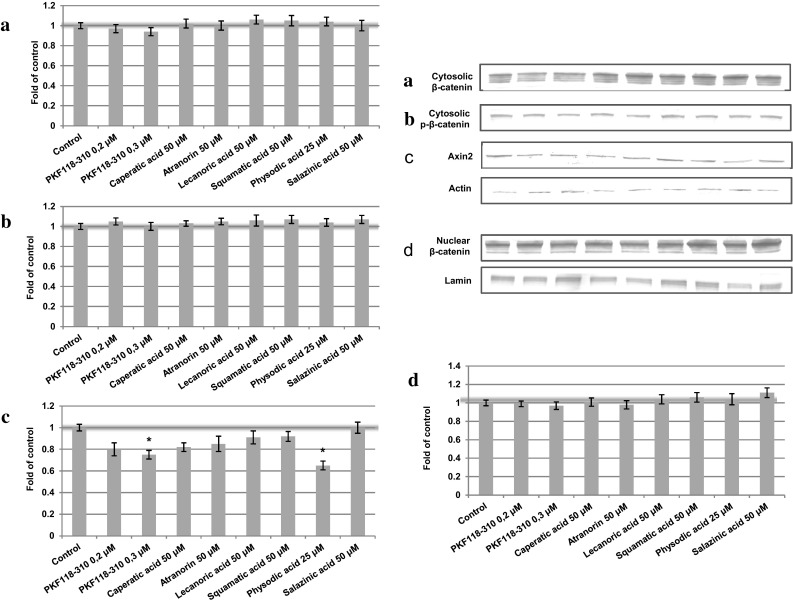
The effect of lichen-derived chemicals on the cytosolic level of β-catenin (**a**) and phospho-β-catenin (**b**) and Axin2 (**c**) and the nuclear level of β-catenin (**d**) in HCT116 cells after 48 h incubation. Representative immunoblots are presented above graphs. Data were normalized against the level of β-actin (cytosolic proteins) or lamin (nuclear proteins). Fold of change in relation to control cells treated with the vehicle where protein level was arbitrarily expressed as 1 was calculated and mean values ± SEM from two independent experiments are shown. Asterisk above bars denotes statistically significant changes, *p* ≤ 0.05

**Fig. 5:**
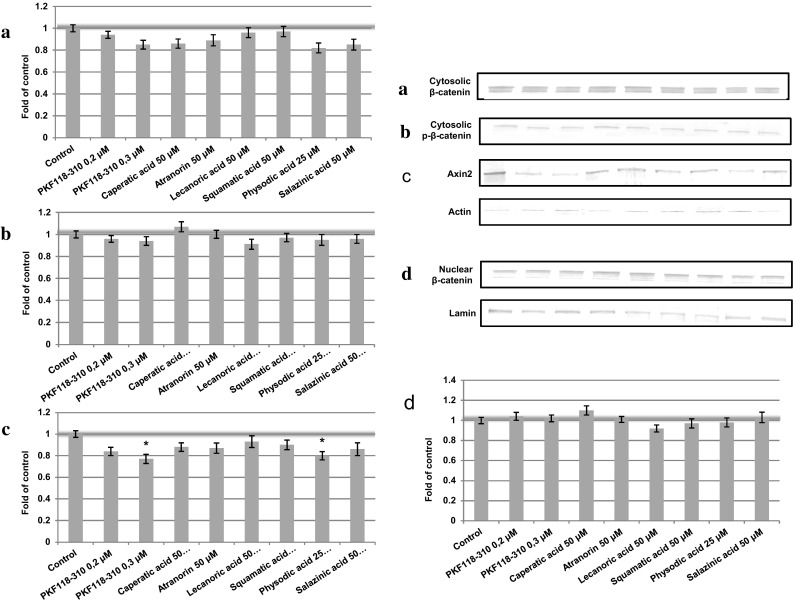
The effect of lichen-derived chemicals on the cytosolic level of β-catenin (**a**) and phospho-β-catenin (**b**) and Axin2 (**c**) and the nuclear level of β-catenin (**d**) in DLD-1 cells after 48 h incubation. Representative immunoblots are presented above graphs. Data were normalized against the level of β-actin (cytosolic proteins) or lamin (nuclear proteins). Fold of change in relation to control cells treated with the vehicle where protein level was arbitrarily expressed as 1 was calculated and mean values ± SEM from two independent experiments are shown. Asterisk above bars denotes statistically significant changes, *p* ≤ 0.05

Additionally, the cytosolic content of Axin2 was assessed in order to verify whether the changes in the transcript level were reflected in the changes in its protein level. The results are shown in Figs. 4d and 5d. Only PKF118-310 and physodic acid reduced the protein level of Axin2 in both HCT116 and DLD-1 cell extracts reflecting gene expression results.

### The effect of lichen compounds on β-catenin level

In order to establish the potential mechanism of action of the tested compounds, we evaluated the cytosolic and nuclear content of β-catenin and the level of β-catenin phosphorylated at Thr41 and Ser45. The cells were incubated in the presence of the chemicals for 48 h. The results are shown in Figs. 4a–c (HCT116 cell line) and 5a–c (DLD-1 cell line). The analysis did not reveal any significant changes in protein content upon the treatment with any of the tested compounds. Neither did any of the compounds affect the level of *CTNNB1* transcript (Fig. 3).

### The analysis of cell cycle and apoptosis

**Fig. 6:**
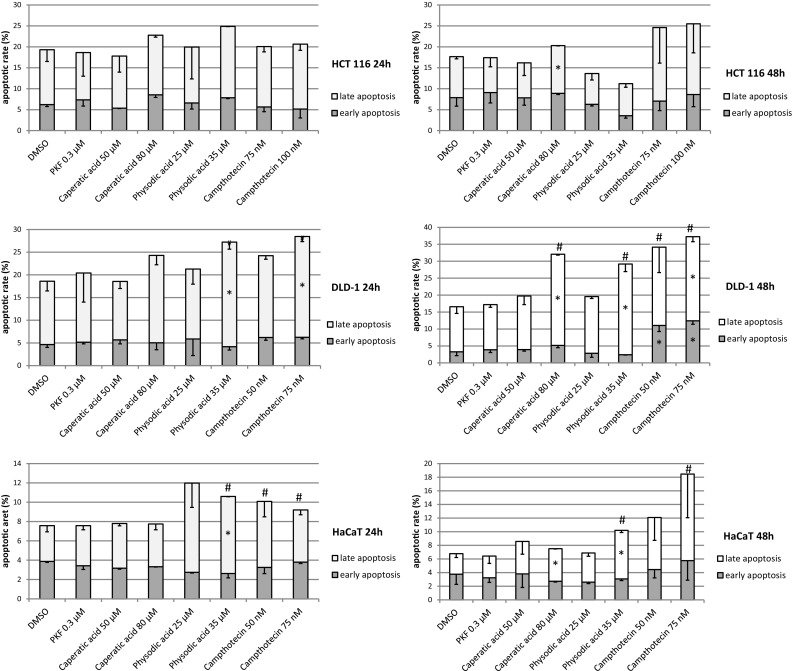
The effect of caperatic acid and physodic acid on cell apoptosis (phosphatidylserine externalization) in HCT116, DLD-1 and HaCaT cell lines after 24 h or 48 h incubation. Mean values ± SD from two independent experiments are shown. Asterisk denotes statistically significant changes, *p* ≤ 0.05. Hash (#) denotes statistically significant changes of the total number of apoptotic cells

**Fig. 7:**
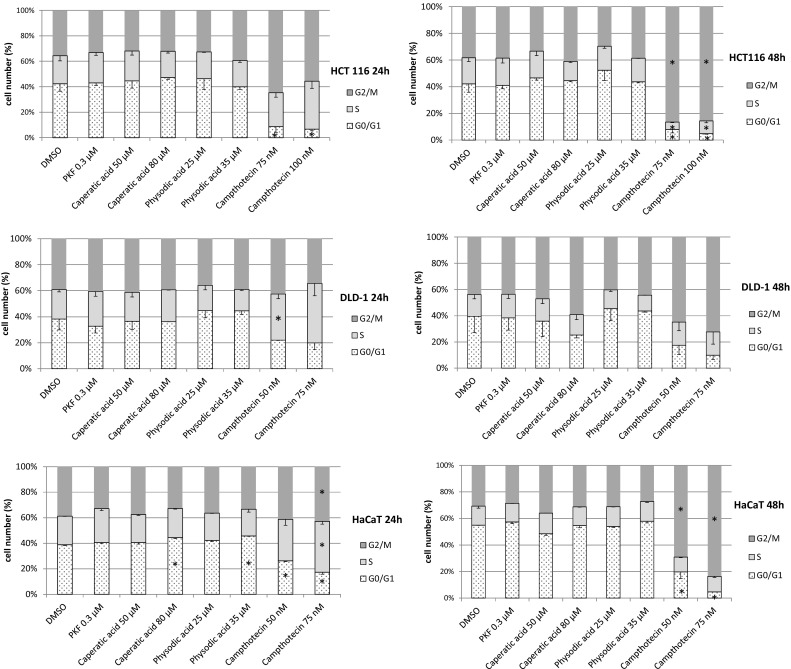
The effect of caperatic acid and physodic acid on cell cycle distribution in HCT116, DLD-1 and HaCaT cell lines after 24 or 48 h incubation. Mean values ± SD from two independent experiments are shown. Asterisk denotes statistically significant changes, *p* ≤ 0.05

We selected caperatic acid and physodic for further analysis based on their strongest potential to modulate Wnt pathway activity. Since both compounds significantly reduced cell viability, although at different concentrations, we wanted to assess whether this can be attributed to increased cell apoptosis. The analysis of phosphatidylserine externalization (Fig. 6) revealed that the higher concentrations of these compounds can moderately increase the rate of apoptosis, especially in DLD-1 and HaCaT cell lines. On the other hand, the compounds did not significantly alter cell cycle distribution in colorectal cancer cell lines or HaCaT cells (Fig. 7). As expected, camptothecin led to cell cycle arrest in the G2/M phase and induced apoptosis; however, it required 48 h incubation.

### Time-dependent effects of caperatic acid and physodic acid on gene expression

**Fig. 8:**
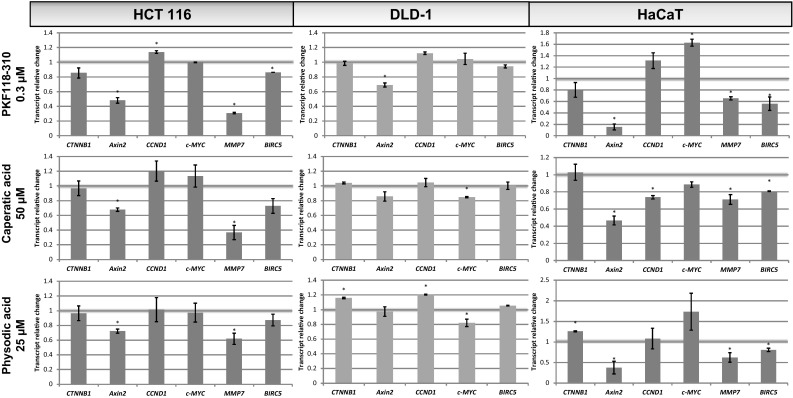
The effect of PKF118-310, caperatic acid and physodic acid on the expression of *CTNNB1* and β-catenin target genes (*Axin2, CCND1, c*-*MYC, BIRC5, MMP7*) in HCT116, DLD-1 and HaCaT cell lines after 24 h incubation. The level of each transcript was calculated in relation to cells treated with the vehicle where expression was arbitrarily set to 1. Mean values ± SD from two independent experiments are shown. Asterisk above bars denotes statistically significant changes, *p* ≤ 0.05

Caperatic acid and physodic acid were selected for further analysis based on their ability to impair *Axin2* expression in all the tested cell lines. In order to better describe their potential to modulate β-catenin-dependent transcription, we assessed the level of β-catenin target genes after 24 h of incubation in the presence of 50 µM caperatic acid or 25 µM physodic acid. For reference, PKF118-310 at the concentration of 0.3 µM was used. The results are shown in Fig. 8. Again, DLD-1 cells were less responsive to the modulatory activity of the investigated chemicals. PKF118-310 reduced the level of *Axin2* transcript by ~30%, but did not affect the expression of the other tested genes which was comparable to its effects in DLD-1 cells after 48 h incubation. Caperatic acid showed a tendency to reduce *Axin2* expression (*p* ≤ 0.1) and reduced *c*-*MYC* transcript by ~15%. On the other hand, physodic acid slightly increased the expression of *CTNNB1* and *CCND1* but reduced the expression of *c*-*MYC*, transcription of which was diminished by 18%.

The inhibitory effects of the compounds were more enhanced in HCT116 cells. The results revealed that PKF118-310 significantly decreased *Axin2* expression and potently reduced the expression of *MMP7*. Additionally, the expression of *BIRC5* (*survivin*) was moderately decreased. The expression of *CCND1* was slightly increased by PKF118-310 treatment. Both caperatic acid and physodic acid diminished *Axin2* expression. Additionally, caperatic acid was much more potent than physodic acid in reducing the level of *MMP7* transcript. The expression of *BIRC5* (*survivin*) also tended to slightly decrease after the treatment with both chemicals.

The immortalized keratinocyte HaCaT cell line was also susceptible to the modulatory effects of the compounds. PKF118-310 showed a very potent reduction in the level of *Axin2*. Moreover, it significantly reduced the expression of *MMP7* and *BIRC5*. Both caperatic and physodic acid led to a decrease in the level of *Axin2*, *MMP7,* and *BIRC5* transcripts. Additionally, caperatic acid reduced the level of *CCND1* in HaCaT cells.

### The effect of caperatic acid and physodic acid on cell migration and invasion

**Fig. 9:**
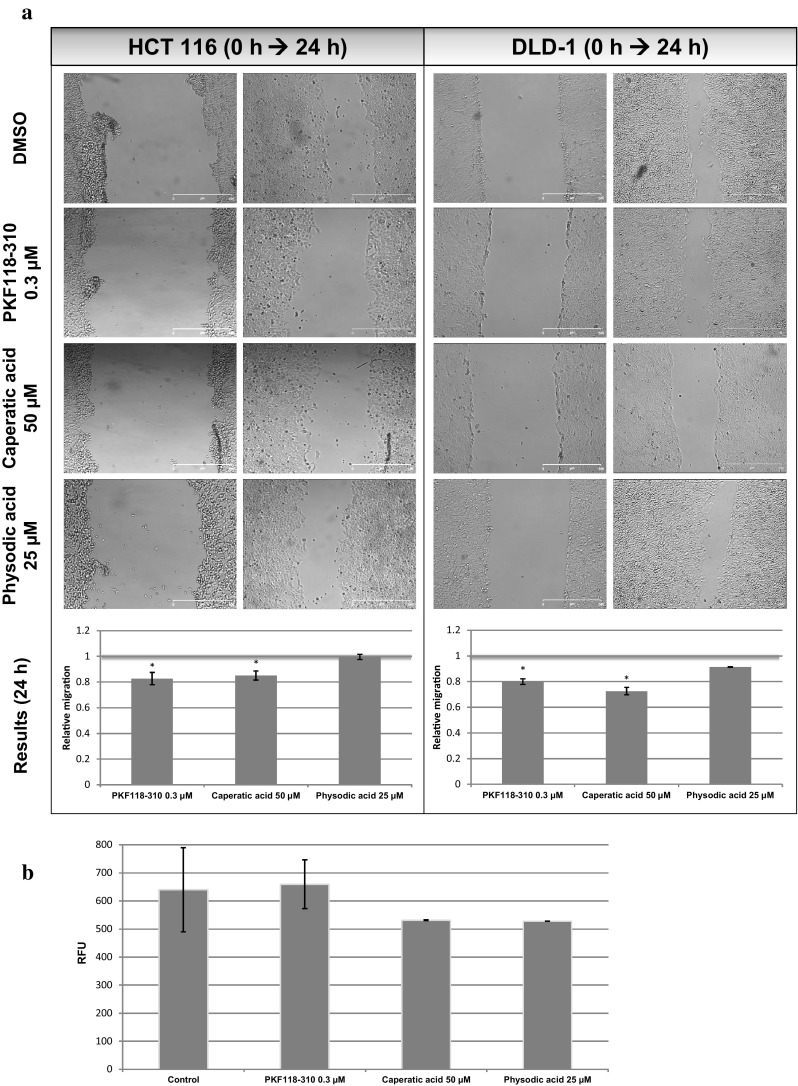
The effect of PKF118-310, caperatic acid and physodic acid on cell migration. **a** The results of the wound healing assay in DLD-1 and HCT116 cells. Relative cell migration was calculated by comparing the change in the area covered by cells immediately after the formation of a scratch and after 24 h between control cells and cells treated with the tested compounds. Mean values ± SD from two independent experiments are shown. Asterisk above bars denotes statistically significant changes, *p* ≤ 0.05. **b** The results of a transwell migration assay with HCT116 cells. The number of invading cells was assessed after fluorescent staining of the cells which migrated through the basement membrane. Mean fluorescence values (relative fluorescent units—RFU) from three experiments ± SD are shown

In order to better describe the anticancer potential of the compounds, we assessed their effect on cell migration using the wound healing (scratch) assay and the results are presented in Fig. 9a. Caperatic acid led to an inhibition of cell migration by ~20%, similarly as PKF118-310. Physodic acid did not show any significant changes in the cell migration potential.

Moreover, since caperatic acid and physodic acid reduced the expression of *MMP7*, we decided to evaluate their effect on the invasive potential of HCT116 cells, which showed higher susceptibility towards the chemicals. The results are presented in Fig. 9b. Both compounds tended to reduce by ~17% of the number of invading cells after 48 h incubation, although the changes were not statistically significant. PKF118-310 did not affect the cell invasive potential what may be consistent with its lack of effect on *MMP7* expression at this time point.

## Discussion

Lichens comprise a large group of diverse species known to produce many secondary metabolites like aliphatic acids, depsides, depsidones, depsones, dibenzofurans, anthraquinones, chromones, xanthones etc. Lichens have been used in traditional medicine for the treatment of wounds, skin problems, and upper respiratory and gastrointestinal symptoms [21]. Many investigations reported the antioxidant, antibacterial, antiviral, antipyretic, anti-inflammatory, and cytotoxic activity of lichen extracts [2]. In our study *Platismatia glauca* was used as the source of atranorin and caperatic acid. Salazinic acid and squamatic acid were derived from *Parmelia sulcata* and *Cladonia uncialis*, respectively, while physodic acid was isolated from *Hypogymnia physodes*. Lecanoric acid was obtained from *Hypocenomyce scalaris*. Extracts of these lichen species were shown to possess antioxidant, antimicrobial, genotoxic, cytotoxic, and pro-apoptotic (anticancer) effects in breast and colon carcinoma cell lines [7, 22–24]. Although several lichen extracts have been shown to inhibit colon carcinogenesis [8, 13, 14], the mechanisms of the anticancer activity of either extracts or single isolated compounds remain largely unknown. The aim of this study was thus to partly fill this gap.

In the first step, we evaluated the cytotoxic effects of the chemicals with the use of the MTT assay. All the compounds dose-dependently reduced the viability of both HCT116 and DLD-1 colon carcinoma cells with the exception of lecanoric acid in DLD-1 cells. Physodic acid showed enhanced cytotoxicity at significantly lower concentrations than other chemicals; however, atranorin and caperatic acid also induced strong cytotoxic effects at the highest concentration applied. Thus, the concentration of physodic acid used in further treatments was lower than that of other compounds. Our results are in agreement with published literature. Previous studies on physodic acid investigated the cytotoxic properties of this depsidone against different cancer cell lines. Strong cytotoxicity was observed towards breast cancer (T-47D, MCF-7, and MDA-MB-231), human colon carcinoma (LS174), and human melanoma (FemX and A375) cells [7, 9, 10]. Interestingly, physodic acid showed selective cytotoxicity towards breast cancer cells, in comparison to non-tumorigenic cells (MCF-10A) [7]. The results of our study do not confirm such selectivity, as HaCaT cells were as susceptible to viability reduction by physodic acid as colorectal carcinoma cell lines. Other lichen compounds tested in our experiments showed weaker cytotoxic activity than physodic acid in all the cell lines. Moreover, the rest of the compounds (except squamatic acid) led to a milder decrease in viability in HaCaT keratinocytes, suggesting a certain degree of selectivity towards cancer cells. The results of our study, as well as the data presented by other authors, demonstrate that physodic acid, next to usnic acid [25], is a compound with potent cytotoxic activity against cancer cell lines. Moreover, published data have proven its pro-apoptotic properties [9]. Physodic acid stimulated apoptosis due to a reduction in Bcl2 level and stimulation of Bax expression and caspase-3 activity in A375 melanoma cells [10]. The stimulation of apoptosis in DLD-1 cells and HaCaT cells was also observed in our study. The stronger cytotoxic effects of physodic acid may be at least in small part related to its ability to lead to the downregulation of *BIRC5* expression which encodes the anti-apoptotic survivin. The exact mechanisms of the induction of apoptosis by physodic acid in colorectal cancer cells require further elucidation.

It is widely accepted that the initiation and progression of colorectal carcinogenesis is significantly associated with aberrations in Wnt signaling. The canonical Wnt pathway is responsible for the control of cell proliferation, migration, and cell death by regulating the transcriptional activity of β-catenin through blocking its cytosolic sequestration. In physiological states, β-catenin-mediated transcription is activated by the presence of Wnt ligands which act on cell membrane receptors and block the activity of cytosolic protein complex consisting of APC, GSK3β, Axin, and casein kinase which is responsible for the stimulation of β-catenin proteasomal degradation. After nuclear translocation, β-catenin interacts with TCF/LEF transcription factors and induces the expression of genes responsible for the regulation of cell cycle (*CCND1*, *c*-*MYC*), cell migration (*MMP*-*7*), and apoptosis (*BIRC5*) and of other regulators including *Axin2*. Around half of CRC patients bear inactivating mutations in the *APC* gene. Also mutations in *CTNNB1* gene encoding β-catenin and other pathway-related genes contribute to Wnt pathway aberrant activation. The importance of Wnt dysregulation in colon carcinogenesis makes it a promising therapeutic target. Several synthetic chemicals like sulindac [26] or a group of quinazoline compounds [27] were able to exert anticancer effects in colon carcinoma cell lines via inhibition of Wnt signaling. There is also evidence that structurally diverse bioactive food components may have similar effects [28].

The action of lichen secondary metabolites may be mediated by the modulation of cell signaling pathways. In this regard, atranorin (100-200 µM) induced p38 and Bax and led to cell cycle arrest and apoptosis induction in colorectal carcinoma HT-29 cells [8]. Additionally, atranorin and lecanoric acid (25-50 µM) but not structurally similar squamatic acid were able to reduce AhR-mediated XRE-dependent *CYP1A1* gene expression [12]. On the other hand, salazinic acid and squamatic acid did not significantly affect the phosphorylation of ERK1/2 or Akt in human cancer cells [6]. Since several lichen extracts were shown to inhibit colon carcinogenesis which is frequently associated with disruptions in Wnt signaling, we wanted to elucidate whether the studied lichen chemicals are able to modulate this pathway in colon carcinoma cell lines.

Of all the analyzed chemicals in our study, only caperatic acid and physodic acid showed β-catenin-dependent transcription inhibition comparable to the action of the known β-catenin/TCF4 antagonist—PKF118-310, which was identified as a β-catenin inhibitor over a decade ago [29]. Although PKF118-310 effectively blocked β-catenin-dependent transcription in all the tested cell lines what was reflected by the decrease in the level of *Axin2* transcript and protein, DLD-1 cells were more resistant to the inhibitory effects of lichen chemicals. The genetic profile in these cell lines is different. Among many other alterations, DLD-1 cells bear the mutation in *APC* gene while HCT116 cells are characterized by a monoallelic *CTNNB1* activating mutation. It will require further elucidation what factors determine the Wnt inhibitory response exerted by these chemicals. The activity of PKF118-310 and caperatic acid was highest after a 24 h incubation period and tended to decline with longer incubation time in contrast to physodic acid whose inhibitory effects appeared to be strongest after 48 h treatment. This indicates a possibility that caperatic acid and physodic acid may effectively modulate Wnt-driven carcinogenesis. On the other hand, both caperatic and physodic acid affected the function of Wnt signaling in HaCaT keratinocytes what suggests that interference with the activity of this pathway in normal cells may contribute to the appearance of side-effects, e.g., reduced tissue regeneration.

The mechanism of Wnt pathway inhibition (evidenced by the reduction in *Axin2* expression, which is solely targeted by Wnt signaling) by caperatic acid and physodic acid does not seem to be dependent on the attenuation of the nuclear translocation of β-catenin since none of the compounds affected the level of expression or the subcellular localization of this protein. This suggests other mechanisms, possibly operating in the nucleus. Similarly, a diterpenoid henryin has been recently shown to interfere with β-catenin/TCF4 interaction in colorectal cancer cells [30]. In this regard, both caperatic and physodic acid mimicked to a large extent the action of PKF118-310, which reduces the transcriptional activity of β-catenin by blocking its interaction with TCF4. However, other mechanisms of transcription modulation, independent of modulating β-catenin, cannot be excluded. In fact, most naturally active compounds act pleiotropically thus it can be assumed that also other signaling pathways may undergo modulation by these compounds. This will require further study.

Importantly, caperatic acid led to an inhibition of migration of colorectal cancer cells and this effect was similar to the action of PKF118-310. Also, due to the down-regulation of *MMP*-*7* expression by caperatic acid and physodic acid, we hypothesized that these compounds may affect cell invasion as a recent report has shown that the silencing of β-catenin in colorectal cancer cells blocked invasion by reducing MMP-7 and inducing E-cadherin [31]. Thus, we did not evaluate cell invasion in DLD-1 cell line which is known to lack the expression of MMP-7 [32]. Although the effect of caperatic acid on the expression of *MMP*-*7* was higher than of physodic acid, both compounds showed a similar tendency to decrease the invasive potential of HCT116 cells suggesting the engagement of also other factors. Interestingly, PKF118-310 did not affect cell invasion but it might not be surprising since it did not alter the expression of *MMP*-*7* after 48 h incubation. The ability of caperatic acid to diminish both the migratory and invasive potential of colon cancer cells is of utmost importance since cell spreading is associated with significantly worse prognosis in any cancer type. This, on the other hand, raises a possibility of using this compound or its derivatives for therapeutic purposes even in more advanced tumors. Indeed, adjuvant chemotherapy using 5-fluorouracil or oxaliplatin is used after surgery in the treatment of patients with nodal involvement. Probably, the therapeutic effects of caperatic acid would not be satisfactory based on the requirement of using high concentrations. In this regard, physodic acid shows more preferable pharmacodynamics. However, caperatic acid has an even greater potential to be used in colorectal cancer chemoprevention based on its reduced toxicity towards non-cancer cells. This is also based on the fact, that the dysregulation of Wnt pathway activity is detected as early as at the pre-malignant polyp stage. These hypotheses need verification in animal studies. Several other naturally occurring chemicals have been also identified as Wnt pathway inhibitors [28, 33]. Lupeol, silymarin, and grape compounds suppressed colon carcinogenesis through the inhibition of the nuclear translocation of β-catenin and attenuation of the transcriptional activity and expression of its target genes [34–36]. It has been recently proposed that lichen extracts could be applied as food additives making use of their preservative capacity [21]. Bioactive food components are especially beneficial since they often lack any pronounced toxicity. This is true for caperatic acid suggesting that it could be used in the chemoprevention of colorectal carcinogenesis based on its Wnt inhibitory activity.

Taken together, we have characterized the potential of caperatic acid and physodic acid to inhibit the expression of β-catenin-dependent genes and attenuate cell migration. We selected caperatic acid as the best inhibitor of Wnt signaling among the tested lichen compounds which has given its broadest effects on β-catenin-dependent gene expression and taking into account its cancer-specific cytotoxic effects. Further research should elucidate the detailed mechanisms of activity of these compounds and confirm the possible chemopreventive and chemotherapeutic use in other cancer cell and animal models.
